# Predictive Factors of Autologous Bone Marrow-Derived Mesenchymal Stem Cell Therapy’s Success in the Treatment of Perianal Fistulas in Patients With Crohn’s Disease

**DOI:** 10.7759/cureus.88534

**Published:** 2025-07-22

**Authors:** Sofia Oubaha, Oussama Nacir, Imane Zouaki, Khadija Krati

**Affiliations:** 1 B2S Research Laboratory, Faculty of Medicine and Pharmacy, Cadi Ayyad University, Marrakesh, MAR; 2 Gastroenterology and Hepatology, Mohammed VI University Hospital of Marrakesh, Marrakesh, MAR

**Keywords:** autologous, bone marrow, crohn’s disease, mesenchymal stem cells, morocco, perineal fistulas

## Abstract

Introduction

Crohn’s disease (CD)-associated perianal fistulas are a challenging and debilitating complication, often resistant to standard medical and surgical treatments. Mesenchymal stem cell (MSC) therapy, particularly using adipose- or bone marrow-derived MSCs (AMSCs/BM-MSCs), offers a promising regenerative approach, yet data on autologous BM-MSCs remain limited.

Objective

This pilot study aimed to evaluate the safety and efficacy of autologous BM-MSCs in treating perianal fistulas in CD patients, and to identify potential predictive factors influencing treatment outcomes.

Methods

A single-center, prospective, single-blind randomized study was conducted over six months at Mohammed VI University Hospital in Marrakech, Morocco. Twenty CD patients with complex perianal fistulas unresponsive to conventional therapy were enrolled, of whom eight completed the 24-week follow-up. Patients were randomized into two groups: local BM-MSC administration only (CroMaPCs) and combined local plus intravenous administration (CroMaPCs+). Outcomes were assessed clinically and radiologically at Weeks 2, 6, and 24.

Results

At 24 weeks, complete fistula healing was observed in three patients (37.5%), with higher response and healing rates in the CroMaPCs+ group (50%) compared to the CroMaPCs group (25%). No treatment-related adverse events were reported. Factors such as age, immunosuppressive therapy, and combined BM-MSC intravenous administration may have influenced outcomes, although the small sample size limits definitive conclusions.

Conclusion

Autologous BM-MSC therapy appears safe and potentially effective in managing refractory perianal fistulas in CD. Combined local and systemic administration may enhance healing. Larger, controlled trials are needed to validate these findings and refine patient selection and treatment protocols.

## Introduction

Crohn's disease (CD) is a chronic inflammatory bowel disorder that can lead to a variety of complications, including perianal fistulas. These fistulas are one of the most challenging and debilitating manifestations of the disease, significantly impacting a patient's quality of life as they are often refractory to conventional treatments, which include antibiotics, immunosuppressants, and surgical interventions [1]. As such, there is an ongoing search for more effective therapeutic options. Regenerative medicine, particularly mesenchymal stem cells (MSCs), has emerged as a promising candidate for treating CD-related perianal fistulas due to their immunomodulatory properties, ability to promote tissue repair, and potential to modulate inflammation [2-4]. While the efficacy and safety of adipose-derived and allogeneic bone marrow-derived MSCs in treating perianal fistulizing CD have been demonstrated in several studies, showing potential in managing this condition [3,4]. However, there is a lack of data regarding autologous bone marrow-derived MSCs for the same purpose. Furthermore, its success rate varies widely, suggesting that certain factors may influence treatment efficacy [2-4].

The primary aim of this study was to evaluate the efficacy and safety, and to analyze the predictive factors for the success of autologous bone marrow mesenchymal stromal cell (BM-MSC) therapy in the treatment of CD-related perianal fistulas. Identifying these factors could not only help optimize treatment strategies but also guide the selection of appropriate candidates for stem cell therapy, improving patient outcomes and minimizing the risk of failure. By exploring variables such as patient characteristics, perineal disease severity, previous treatments, and the specific procedural details of BM-MSC therapy, we hope that this study will give some valuable insights into the clinical application of stem cell therapy in CD.

## Materials and methods

Study design

This is a monocentric, prospective, pilot study with a descriptive and analytical purpose, using single-blind randomization, conducted over a period of six months starting on May 1, 2022. It focuses on a series of patients followed for Crohn's disease with perineal fistulas at the Hepato-Gastroenterology Department of Mohammed VI University Hospital of Marrakech in Morocco.

Study objectives

The primary objective of this pilot study was to assess the safety of local and general administration of adult autologous BM-MSCs in patients with perianal fistulizing CD, as well as their efficacy by clinical and radiological assessments. The second objective was to analyze the predictive factors for the success of BM-MSC therapy in the treatment of CD-related perianal fistulas.

Study population

Eligible patients for our study were between 18 and 60 years of age, diagnosed with CD with a complex perianal fistula of at least two months duration, with a controlled luminal disease, must have failed previous standard treatments for perianal fistulas, and must have provided signed consent. We have excluded all patients that are younger than 18 or older than 60 years of age, who have not consented to the study, pregnant patients, patients with an active luminal disease or Crohn’s Disease Activity Index (CDAI) index of more than 220, with treatment naïve fistulas, with a severe anemia, with collections greater than 2 cm, stenosis, or severe proctitis or with fistulas other than perianal fistulas.

Among the 550 patients with chronic inflammatory bowel disease (IBD) followed in our department, 215 were diagnosed with CD, 43 of whom had associated perianal lesions (20% of the patients). In this subgroup, 36 had single or multiple anal fistulas, of which 30 met the inclusion criteria for our study, forming our target population. To select participants for our study, we chose a sample of 20 individuals from the 30 who met the inclusion criteria. Eight patients completed the 24-week post-injection follow-up, providing sufficient data to evaluate the effectiveness of the procedure. Two patients are currently at W0 (Week 0) of the injection, six patients are at W2, and four patients are at W6, with results still being collected. The eight patients included in our study were then randomized 1:1 into two groups through single-blind randomization: CroMaPCs (only local injection of BM-MSCs into the perianal fistula’s tract) and CroMaPCs+ (local injection of BM-MSCs into the perianal fistula’s tract and intravenously).

The diagnosis of CD was confirmed as per the European Crohn's and Colitis Organisation (ECCO) guidelines on a set of clinical, biological, radiological, endoscopic, and/or histological arguments. We classified patients into phenotypes using the Montreal classification, which includes age at onset, location, and behavior of the disease. For the classification of perianal lesions, we used the Cardiff classification. Pelvic MRI was done to evaluate the presence and the complexity of perianal fistulas and of any perianal abscess or collection, whenever possible. For the evaluation of luminal activity, the CDAI score was used, which includes multiple variables: number of liquid stools, abdominal pain, general status, presence of extraintestinal complications, use of antidiarrheal drugs, presence of an abdominal mass, body weight, and hematocrit.

Collection of mesenchymal stem cells

**Figure 1:**
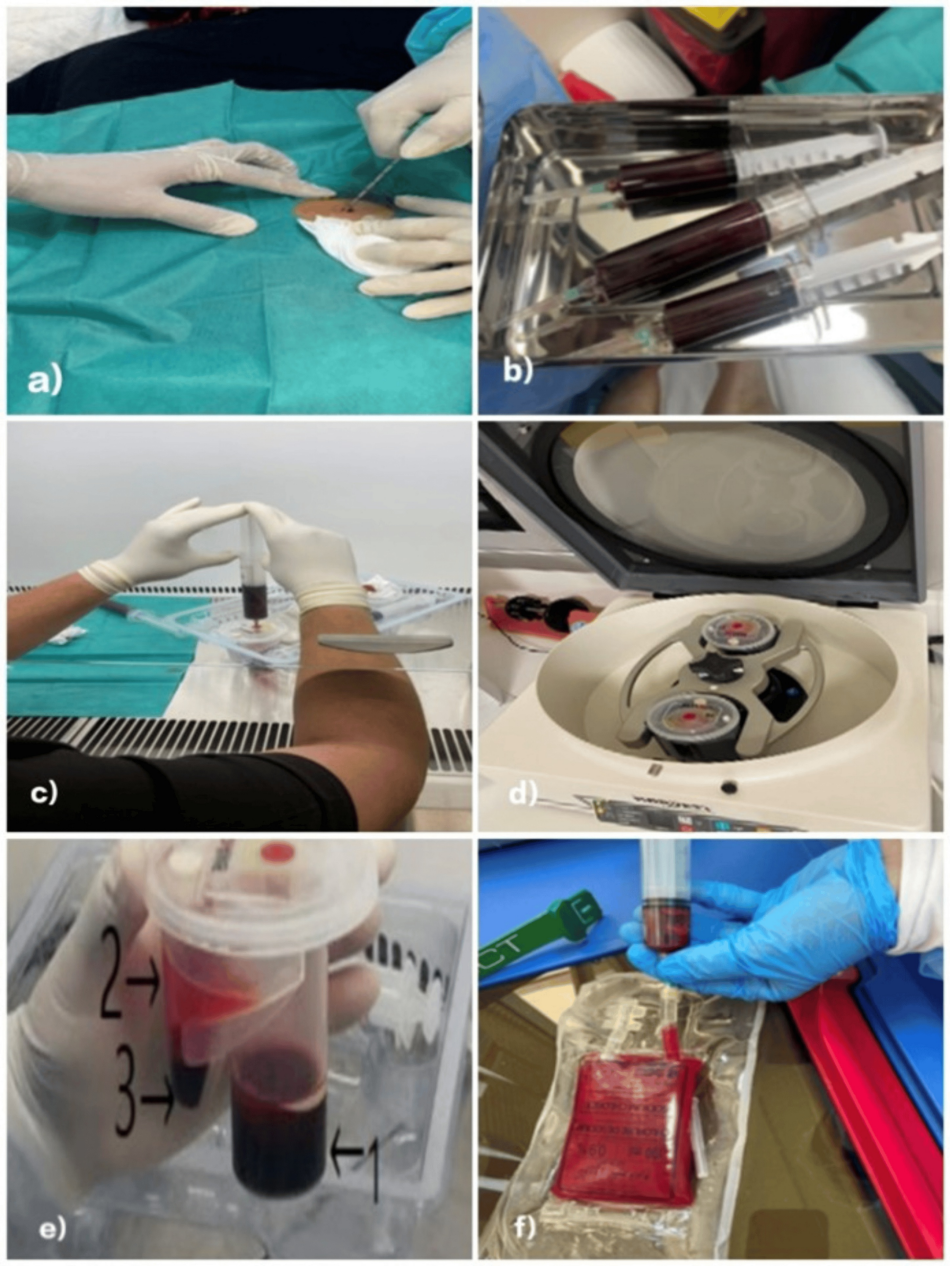
Steps of the process of autologous bone marrow mesenchymal stromal cells (BM-MSCs) collection in our study a) Introduction of the Jamshidi needle at the level of the posterior iliac crest. b) Syringes with the aspirated bone marrow. c) Placement of the aspirate in the marrow chamber. d) Centrifugation of the aspirate. e) Concentration of hematopoietic stem cells derived from bone marrow (1 - blood, 2 - plasma, 3 - bone marrow concentrate). f) Preparation of the stem cell infusion.

In our study, we prioritized bone marrow as the source for MSC extraction. Thus, we have conducted bone marrow aspirations on all patients included in the study (Figure 1). This aspiration was performed at the level of the posterior iliac crest using a single-use kit consisting of a Jamshidi trocar, aspiration syringes, sterile gauze, blades, and intravenous catheters in strict sterile conditions. With local anesthesia, approximately 60 mL of bone marrow per patient was aspirated into heparinated syringes, ensuring that the trocar is rotated during aspiration to vary the areas of collection. The syringes were then treated in the Regenerative Medicine Centre of our University Hospital in Marrakech in order to isolate the MSCs. The aspirated product is placed into a device containing a filter to remove bone debris from the sample, then placed in the marrow chamber to be centrifuged at 400 g for 10 minutes to separate mononuclear cells. After centrifugation, the excess plasma is removed using a syringe. Then, another syringe is used to resuspend the bone marrow concentrate and the remaining plasma to obtain a product ready for injection. A 1-2 mL sample of the centrifuged aspirate is collected for flow cytometry analysis. Aspiration and preparation of BM-MSCs are shown in Figure 1.

Administration of mesenchymal stem cells

Before administration, MSCs were characterized according to the criteria defined by the International Society for Cellular Therapy (ISCT). The cells expressed the typical surface markers CD73+, CD90+, and CD105+, and were negative for CD14, CD34, CD45, and HLA-DR, as confirmed by flow cytometry. Cell viability was assessed using the trypan blue exclusion method and exceeded 95% before injection. All MSC preparations were tested for sterility (aerobic and anaerobic bacteria, fungi), mycoplasma, and endotoxins prior to administration, in accordance with GMP regulations.

**Figure 2:**
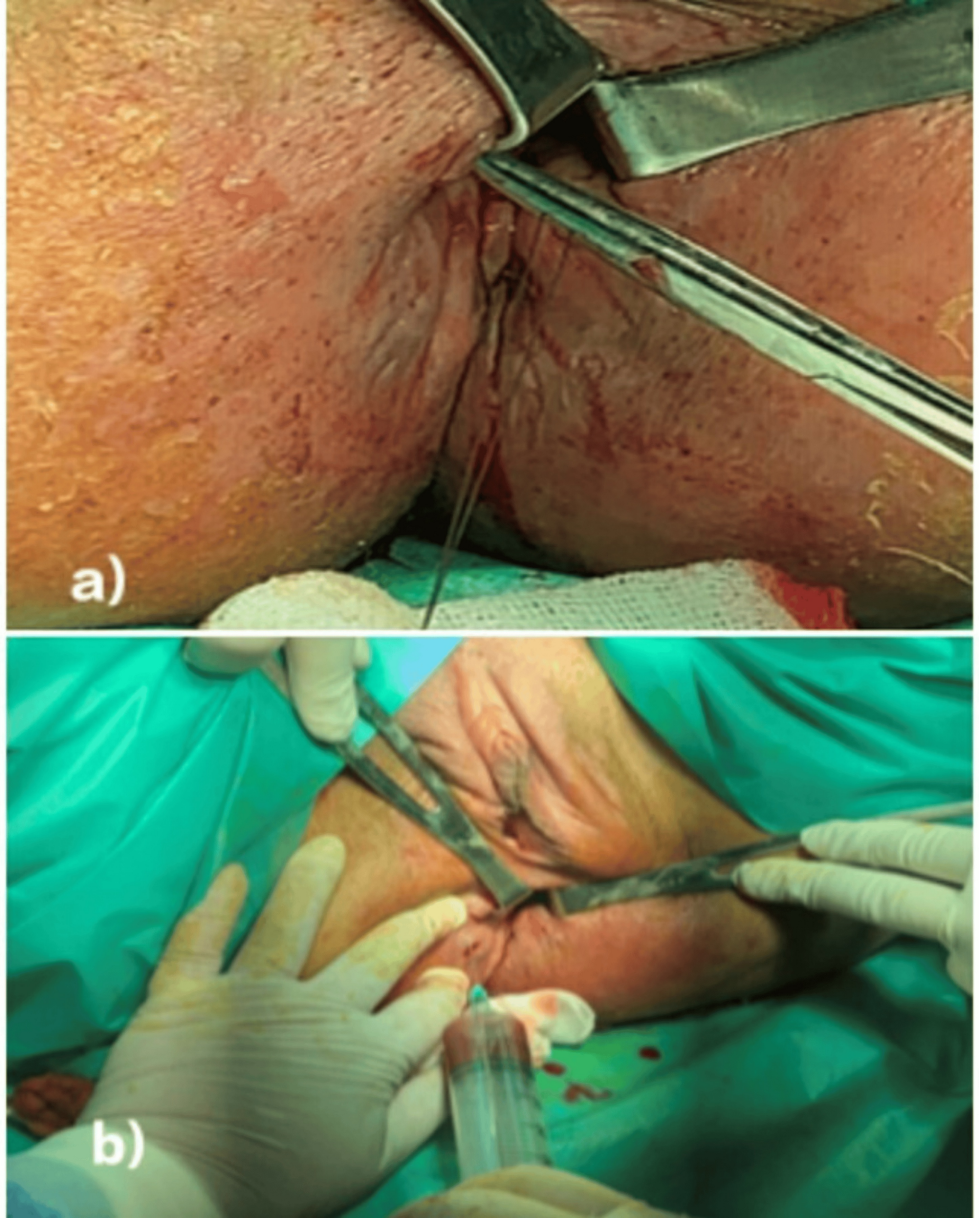
Steps of the local administration of the autologous bone marrow mesenchymal stromal cells (BM-MSCs) a) Closure of the internal fistula opening with a 2-0 Vicryl suture. b) Injection of stem cells into the fistula tracts.

Before injection, the entire fistulous tract must be curetted, then the internal orifice is closed using resorbable sutures. The volume of injected MSCs was adapted to the size and number of fistulous tracts, as measured intraoperatively using a flexible probe. The total dose ranged from 20 to 60 million cells per patient, depending on the fistula burden. The injection was performed circumferentially into the submucosa and fistulous wall, around the internal opening. The mean volume injected per tract was approximately 1 mL per cm of tract length, with a maximum of 4 mL per fistula. Among the eight patients included and after randomization, four patients received an injection of MSCs at the fistulous tracts only (CroMaPCs), while the other four patients received an injection of MSCs at the fistulous tracts along with an intravenous infusion (CroMaPCs+). Administration of BM-MSCs is shown in Figure 2. Patients were admitted to the hospital for 48 hours after administration to monitor for acute local or systemic side effects.

Initial assessment and follow-up

The healing was assessed using clinical criteria, particularly the absence of pus discharge upon light digital pressure and the re-epithelialization of the fistula openings. Meanwhile, in one patient, complete healing was observed in three out of five fistula openings. The initial visit consisted of a thorough clinical examination, biological evaluation, rectosigmoidoscopy or colonoscopy, and a pelvic MRI. The injection of BM-MSC was then administered within two weeks following the initial visit. The follow-up protocol was as follows: a clinical evaluation at W2, then a clinical and biological assessment at W6, and finally a clinical, biological, and, if possible, a morphological evaluation (pelvic MRI) at W24. Fistula remission was defined as complete closure of all external openings and no collections larger than 2 cm on pelvic MRI. Fistula response was defined as closure of more than 50% of all openings or a decrease in fistula discharge by ≥50%.

Ethical considerations

The study was conducted in compliance with the Declaration of Helsinki and in accordance with “Guidelines for Stem Cell Research and Therapy” by our Department of Regenerative Medicine and after approval from our Institution's Ethics Committee. Patients were informed about the aim of the study and their right to withdraw at any stage of the study. Data were collected with respect to the anonymity of the patients. Informed written consent was obtained in both French and Arabic before the study began.

Data collection and analysis

Data collection was based on the completion of exploitation forms, gathering necessary information from the medical records, and the hospital's computerized system. The collected data included the following variables: demographic data (age, gender, profession, origin, and place of residence), clinical data (personal and family history, time of diagnosis of Crohn's disease with perianal fistula, mode of presentation, surgical history since diagnosis, and disease topography), biological data, radiological data (pelvic MRI), endoscopic data, histological data, Montreal classification, Cardiff classification, activity score (CDAI), therapeutic data, and evolutionary data. For data analysis, we used Microsoft Excel 2016 (Microsoft® Corp., Redmond, WA) and SPSS (IBM SPSS Statistics for Windows, IBM Corp., Version 22, Armonk, NY). The study included descriptive analysis, calculating averages for quantitative variables and frequencies and percentages for qualitative variables.

## Results

The average age of our patients was 40.75 years, with extremes ranging from 23 to 62 years and a predominance in the age range of 30-50. The average age of patients in the CroMaPCs+ arm was younger, at 30.7 years, than in the CroMaPCs arm, at 44.25 years. A slight male predominance was noted in our study, with a sex ratio M/F of 1.66. All patients in our study are from urban areas and reside there. The majority of patients were unemployed (75%), one patient is a student (12.5%), and one is a civil servant (12.5%). No specific medical history was noted in two-thirds of the patients. However, treated pulmonary tuberculosis was reported in one patient, a major depressive disorder, and a sibling with CD in one patient, and recurrent urinary infections and stillbirth in one patient. No cases of type 2 diabetes or hypertension were recorded in our series. There was one case of a passive smoker and no history of active smoking or alcohol consumption for the rest. None of our patients have undergone digestive surgery or any other surgery before the diagnosis of CD.

In 50% of the cases, the diagnosis of the perianal fistula was made in the same year as the diagnosis of CD. In the other 50% it was diagnosed after three years of the diagnosis in three patients and 29 years in one case. Crohn's disease was revealed by a moderate flare-up in the majority of cases included in the study (five patients), a severe flare-up in one patient, perineal fistulas in one patient, and rectal bleeding in one patient. In our case series, five patients underwent surgical interventions for various indications during the course of their disease; two patients for fistula drainage with placement of a drainage seton, one for fistula drainage then fistulectomy, one for fistulectomy and a right hemicolectomy, and one for a diverting colostomy.

The topography of the CD involvement was ileocolonic in four patients, colonic in three patients, and rectal in one patient. According to Cardiff’s classification, three patients (37.5% of cases) had superficial fissures (U1) classified as U1a (anterior), U1b (lateral), and U1c (with skin tags). All of the patients in our study had fistulas and were classified as follows: five had an F1a, one had an F1b, one had an F2a, and one had an F1c2c. None of the patients included in our study had stenosis (S0). As for the Montreal classification, seven patients were classified as A2, and one patient was classified as A3 according to their age. In our series, five patients are classified as L2, two patients are classified as L3, and one patient is classified as L1. We recorded three patients classified as B3p, two patients classified as B1p, and three patients classified as B2, B1, and B2B3p, respectively. Six of our patients (75%) had mildly active disease with CDAIs ranging from 154 to 210, with an average of 175.8. Two patients were in remission with CDAIs below 150.

As for biological findings, blood count was normal in five patients (62.5%) while it was abnormal in three patients (37.5%). One case presented moderate anemia at 9.9 g/dL, one had neutrophilic leukocytosis, and one had lymphopenia. Hemoglobin ranged from 9.9 to 15.7 g/dL, with an average of 12.7 g/dL. The C-reactive protein was normal in five patients (62.5%) and moderately elevated in three patients.

In our series, the results of the colonoscopy are distributed as follows: internal rectal fistula openings were found in three patients, erythematous colonic mucosa was found in two patients, erythematous rectal mucosa was found in two patients, superficial geographic ulcerations with areas of healthy mucosa were found in three patients, aphthous ulcerations were found in two patients, pit-shaped ulcerations were found in two patients, contact bleeding was found in two patients. Pelvic MRI was initially performed in six patients (75%). The results were as follow: anoperineal fistula tracts were found in all the patients who underwent pelvic MRI, rectal wall thickening was found in two patients, necrotic inguinal lymph nodes were found in two patients, a pelvic abscess was found in three patients, ileal loop enlargement in the ileocecal region was found in two patients, fat infiltration in the pelvic region was found in one patient, a complex uterovaginal-anal fistula was found in one patient. Seven patients out of the eight included in the study underwent an enteric CT scan. The results are as follows: rectal wall thickening in two patients, right colon wall thickening in one patient, left colon wall thickening in one patient, thickening of the last ileal loop in two patients, neighboring sclerolipomatosis in two patients, mesorectal fat infiltration in two patients, and fistula tracts identified in two patients.

Before the study period, all patients received different medical treatments that were modified several times during the course of the disease for better control. The previous treatments of all patients are detailed in Table 1. During the study period, two patients were under combotherapy of Infliximab (IFX) and Azathioprine (AZA), four under AZA only (three at 2.5 mg/kg/day and one at 2 mg/kg/day), one under Methotrexate, and one under Adalimumab (ADA).

**Table 1:** Demographic and clinical characteristics of patients in our study 5-ASA: 5-Aminosalicylic Acid; 6MP: 6-Mercaptopurine; ADA: Adalimumab; AZA: Azathioprine; CD: Crohn’s Disease; F: Female; IFX: Infliximab; M: Male; MTX: Methotrexate

Groups	CroMaPCs+				CroMaPCs			
Patient code	Patient 1	Patient 2	Patient 3	Patient 4	Patient 1	Patient 2	Patient 3	Patient 4
Age/sex	38/M	39/F	28/M	44/M	39/F	62/M	53/F	23/M
Disease duration (years)	4	13	4	5	2	33	4	4
Disease location	Ileocolonic	Rectal	Ileocolonic	Colonic	Colonic	Ileocolonic	Colonic	Ileocolonic
Surgical history	Placement of drainage setons	Diverting colostomy	No	Placement of drainage seton	Fistulectomy, placement of drainage setons	Right hemicolectomy, fistulectomy	No	No
Past medical treatment	5-ASA, IFX	AZA, 6MP, MTX, IFX	AZA	5-ASA, AZA, MTX	AZA	AZA, IFX	AZA	AZA, 6MP, IFX, ADA
Current medical treatment	IFX + AZA	AZA 2.5 mg/kg/day	AZA 2 mg/kg/day	MTX	AZA 2.5 mg/kg/day	IFX + AZA	AZA 2.5 mg/kg/ day	ADA
Baseline CDAI	166	180	210	176	154	196	168	157

After the BM-MSC injection, in W2, none of the cases presented complete healing. This was for both arms. CroMaPCs and CroMaPCs+ had healing and response rates of 0%. At W6, after the injection, the healing rate was still null in both arms; however, the response rate was at 25% (two cases) in the CroMaPCs+ arm: one with a persistent external fistula opening (EFO) but no longer draining pus and one with a persistent EFO that releases <50% of its initial discharge in pus under pressure.

**Figure 3:**
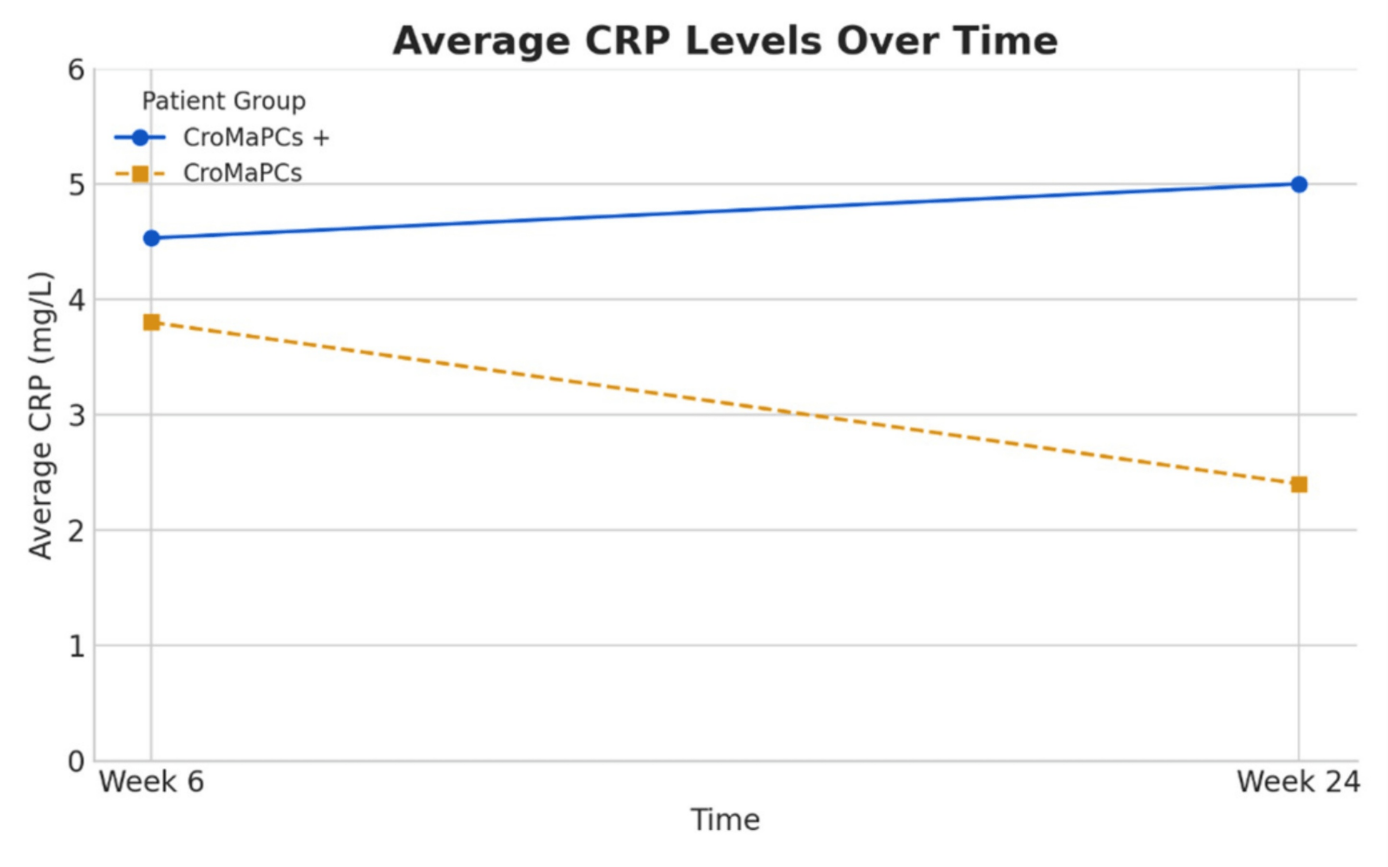
Evolution of the average C-reactive protein between the two groups over time

At W24, complete healing of all perianal fistula openings was observed in three out of eight patients (37.5%); two of them are in the CroMaPCs+ arm (50% of this group) and one in the CroMaPCs arm (25% of this group). The fistula response rate was also 37.5%; two cases from the CroMaPCs+ arm (50% of this group) and only one in the CroMaPCs arm (25% of this group). No adverse effects related to the injection of MSCs were reported in any of our cases. The clinical evolution of our patient is also summarized in Table 2, as well as the biological evolution of the C-reactive protein in Figure 3.

**Table 2:** Clinical outcomes timeline

Group	Patient	Number of fistula(s)	W2 status	W6 status	W24 status
CroMaPCs+	Patient 1	3	Pus discharge	Healing, no discharge	Healing, no discharge
	Patient 2	1	Pus discharge	Healing, no discharge	Healing, no discharge
	Patient 3	1	Serous discharge	Healing, serous discharge	Complete healing
	Patient 4	3	Pus discharge	Healing, <70% of pus discharge	Complete healing
CroMaPCs	Patient 1	2	No discharge	Healing, no discharge	Healing
	Patient 2	5	No discharge	Healing, no discharge	2/5 healed
	Patient 3	4	No discharge	Healing, no discharge	Complete healing
	Patient 4	1	Pus discharge	Pus discharge	Pus discharge

## Discussion

Analysis of results in terms of safety and efficacy in comparison to the literature

The use of stem cells in treating CD-related perianal fistulas is based on their capacity for healing. Stem cells not only enhance local cell regeneration through their multipotency, but they also help reduce inflammation and support the repair of damaged perianal tissue, owing to their immunomodulatory properties [1]. MSCs can be derived from either bone marrow, adipose tissue, or other tissues (e.g., placenta). Depending on the clinical requirement, they may be sourced from the patient (autologous) or from a donor (allogenic), and they are being studied in both local and general treatment in CD. The efficacy and safety of adipose-derived and allogeneic bone marrow-derived MSCs in treating perianal fistulizing Crohn's disease have been demonstrated in several studies, showing potential in managing this condition [2-4]. However, there is a lack of data regarding autologous bone marrow-derived MSCs for the same purpose.

The present study demonstrates that autologous BM-MSC therapy is safe in patients with CD having complex perianal fistulae after failure of conventional medical and surgical therapies, which is in accordance with the literature’s findings so far [5,6]. The local administration of MSCs, of any origin (adipose or bone-marrow derived, autologous or allogeneic), has been proven to be safe, with no significant increase in adverse effects compared to placebo in clinical trials [3,4]. In a large RCT (ADMIRE-CD) comparing local administration of adipose-derived MSCs (AMSCs), 17% of patients receiving AMSCs experienced treatment-related adverse events, compared to 29% in the placebo group at 24 weeks [7]. The most common adverse event was a perianal abscess, which was deemed unrelated to MSC therapy and attributed to the manipulation of perianal tissues. In the two follow-up studies of the same study, at 104 and 156 weeks, the profile of safety did not significantly change, with no new or hazardous events related to the treatment [8,9]. In a placebo-controlled trial assessing local administration of allogenic BM-MSCs with three different doses compared to placebo, no serious adverse events were reported, except for one perianal abscess in each group. Additionally, one patient with a family history of colorectal cancer developed cecal carcinoma after receiving MSCs, but this was considered unlikely to be related to the stem cell therapy. In the recent phase I/II clinical trial by Swaroop et al., no serious adverse events were observed in patients receiving allogeneic BM-MSCs in adult patients with perianal fistulizing CD [10]. Studies involving autologous BM-MSCs have similarly shown no increased risk of unhabitual adverse events in the two published studies so far [5,6].

In literature, local administration of MSCs has shown statistically significant response/remission in several studies with combined remission rates varying from 20% to 70% [3,4]. In the Phase 3 ADMIRE-CD trial, patients receiving AMSCs achieved combined remission in 50% of cases, compared to 34% in the placebo group at 24 weeks [7]. Sustained remission was also demonstrated in the two follow-up studies of the same trial at 104 and then 156 weeks [8,9]. In the study by Molendijk et al. on allogeneic BMSCs, fistula remission was observed in 60.8% vs. 33.3% in placebo at 24 weeks [11]. While in the trial of Swaroop et al., 70% of patients receiving allogeneic BMSCs showed a response to fistula treatment, and 20% achieved remission at 24 weeks [10]. While all types of MSCs are generally presumed to have similar properties, several studies have revealed significant differences in their immunomodulatory effects. Comparative studies on AMSCs and BM-MSCs have shown notable molecular and clinical differences in efficacy [3,12]. This suggests that there could be potential therapeutic distinctions between AMSCs and BM-MSCs in treating perianal fistulizing CD, which needs further exploration.

On another note, some remain concerned that autologous MSCs from IBD patients may be impaired. In our study with autologous BM-MSCs, the remission rate was 37.5% and the response rate was also 37.5% at 24 weeks. In literature, the local administration of autologous BM-MSCs has demonstrated a significantly higher numerical healing rate compared to that of allogeneic BM-MSCs [3]. However, Ciccocioppo et al. demonstrated that autologous BM-MSCs were a safe treatment for eight patients over 72 months of follow-up and that the fistula relapse-free survival was 88% at one year, 50% at two years, and 37% at four years for this patient group [5]. Consequently, these less favorable long-term efficacy results might be linked to the autologous origin of the cells, but further larger studies are needed to confirm these results.

Analysis of results in light of different factors

Although the sample in our study is too small to be representative and thus lead to any conclusions, the authors find it interesting to note some important observations. Comparing the two groups of our study, the CroMaPCs+ (local and IV administration of BMSC) and CroMaPCs (local administration of BMSC), we noted that the CroMaPCs+ group had better fistula remission with a 50% rate (two cases) vs. 25% in the CroMaPCs group (one case), as well as a better fistula response rate, with 50% rate (two cases) vs. 25%, respectively, at W24. We also noted that in the CroMaPCs+ group, the fistula response was faster with a 50% response rate vs. 0% in the CroMaPCs group at W6. This could infer that the adjunction of general and local administration of BM-MSCs might give better and faster healing in perianal fistulizing CD. In terms of sex, more male patients seem to show better fistula response/remission than female patients in our study, with a 2/1 ratio for both responses; however, our sample was too small and made mostly of male patients. In terms of age, the patients showing response/remission were in different age groups: three in the 30-40, one in the 40-50, and two in the 50-60-year-old age groups, which may showcase that age could be a predictive factor of BMSCs therapy in fistulizing CD. Four patients of our series had previous perianal surgery, one with diverting colostomy, and two patients with placement of a drainage seton had response/remission after BMSCs therapy, all in the CroMaPCs+ group. However, one patient with a drainage seton did not have a response in the CroMaPCs group. Therefore, our study could not conclude any differences in terms of the efficacy of BM-MSCs according to surgical history. In terms of disease location, most of our patients had either colonic or ileocolonic or rectal CD with no isolated ileal disease, so no observation on the effect of disease location on the treatment efficacy was made. In terms of CD treatment, patients with complete remission were on immunosuppressants during the time of the study (two on AZA et one on MTX) as well as the three others showing response (one on AZA alone and two on combotherapy IFX + AZA), which may suggest a better efficacy of BM-MSCs therapy in conjunction with immunosuppressants than with other protocols combotherapy with IFX or others (ADA). However, one patient was indeed under AZA before and during the time of study, and still did not respond to BM-MSCs treatment. Therefore, large prospective randomized trials including patients with different treatment regimens are needed to evaluate the response rate of each protocol.

Limitations of our study

First, our study is monocentric with a small sample size, the majority of which were male, which limits the generalizability of the results. Due to the lack of a control arm, we could not compare the efficacy of MSCs to the standard of care. Furthermore, not all patients underwent surgical drainage or seton placement before receiving stem cell therapy, meaning we could not assess the response when MSCs were combined with drainage. We also did not investigate the mechanistic properties of MSCs, such as measuring inflammatory cytokines in the serum, rectal tissue, or perianal fistula scraping. Lastly, we administered a single dose of MSCs, consistent with previous MSC trials; however, a repeat injection for patients with partial or inadequate responses might be necessary for optimal outcomes as demonstrated in a recent pediatric study found that repeat injections of BM-MSCs after three months, in cases of no response, led to complete clinical and radiological healing in 83% of patients. The mechanistic aspects of BM-MSCs, such as inflammatory cytokines and changes in microbiota, were not assessed in our study due to limited infrastructure, which could have strengthened our conclusions.

## Conclusions

In conclusion, this study provides promising evidence for the safety and potential efficacy of autologous BM-MSCs in treating perianal fistulizing Crohn's disease. Although the sample size was small and the study lacked a control arm, the positive trends observed, particularly in the CroMaPCs+ group, suggest that combining general and local BM-MSCs administration may lead to more rapid and effective healing in perianal fistulizing CD. The age and treatment regimen of patients also appeared to influence the response to therapy, indicating that further exploration of these factors could help optimize treatment strategies. Despite these encouraging findings, the study's limitations, including its single-center design, small sample size, and lack of mechanistic analysis, highlight the need for larger, multicenter randomized trials to confirm the efficacy of autologous BM-MSCs and explore their therapeutic mechanisms more thoroughly. Future research should also investigate the role of repeat MSC injections and the potential synergy with immunosuppressive treatments to enhance clinical outcomes.
